# Association Between Self-reported Importance of Religious or Spiritual Beliefs and End-of-Life Care Preferences Among People Receiving Dialysis

**DOI:** 10.1001/jamanetworkopen.2021.19355

**Published:** 2021-08-04

**Authors:** Jennifer S. Scherer, Kaylin C. Milazzo, Paul L. Hebert, Ruth A. Engelberg, Danielle C. Lavallee, Elizabeth K. Vig, Manjula Kurella Tamura, Glenda Roberts, J. Randall Curtis, Ann M. O’Hare

**Affiliations:** 1 Division of Geriatrics and Palliative Care, Department of Internal Medicine, NYU Grossman School of Medicine, New York; 2 Division of Nephrology, Department of Internal Medicine, NYU Grossman School of Medicine, New York; 3 Department of Spiritual Care, NYU Langone Health, New York; 4 Department of Health Services, University of Washington, Seattle; 5 US Department of Veterans Affairs (VA) Health Services Research and Development Center of Innovation, VA Puget Sound Health Care System, Seattle, Washington; 6 Division of Pulmonary, Critical Care, and Sleep Medicine, Department of Medicine, University of Washington, Seattle; 7 Cambia Palliative Care Center of Excellence, University of Washington, Seattle; 8 Department of Surgery, University of Washington, Seattle; 9 British Columbia Academic Health Science Network, Vancouver, British Columbia, Canada; 10 Division of Geriatrics, Department of Medicine, University of Washington, Seattle; 11 Geriatrics and Extended Care, VA Puget Sound Health Care System, Seattle, Washington; 12 Division of Nephrology, Stanford University Medical Center, Palo Alto, California; 13 Geriatric Research and Education Clinical Center and Division of Nephrology, VA Palo Alto Health Care System, Palo Alto, California; 14 Kidney Research Institute, University of Washington, Seattle; 15 Division of Nephrology, Department of Medicine, University of Washington, Seattle; 16 Hospital Specialty and Medicine Service, VA Puget Sound Health Care System, Seattle, Washington

## Abstract

**Importance:**

Although people receiving maintenance dialysis have limited life expectancy and a high burden of comorbidity, relatively few studies have examined spirituality and religious beliefs among members of this population.

**Objective:**

To examine whether there is an association between the importance of religious or spiritual beliefs and care preferences and palliative care needs in people who receive dialysis.

**Design, Setting, and Participants:**

A cross-sectional survey study was conducted among adults who were undergoing maintenance dialysis at 31 facilities in Seattle, Washington, and Nashville, Tennessee, between April 22, 2015, and October 2, 2018. The survey included a series of questions assessing patients’ knowledge, preferences, values, and expectations related to end-of-life care. Data were analyzed from February 12, 2020, to April 21, 2021.

**Exposures:**

The importance of religious or spiritual beliefs was ascertained by asking participants to respond to this statement: “My religious or spiritual beliefs are what really lie behind my whole approach to life.” Response options were definitely true, tends to be true, tends not to be true, or definitely not true.

**Main Outcomes and Measurements:**

Outcome measures were based on self-reported engagement in advance care planning, resuscitation preferences, values regarding life prolongation, preferred place of death, decision-making preference, thoughts or discussion about hospice or stopping dialysis, prognostic expectations, and palliative care needs.

**Results:**

A total of 937 participants were included in the cohort, of whom the mean (SD) age was 62.8 (13.8) years and 524 (55.9%) were men. Overall, 435 (46.4%) participants rated the statement about religious or spiritual beliefs as definitely true, 230 (24.6%) rated it as tends to be true, 137 (14.6%) rated it as tends not to be true, and 135 (14.4%) rated it as definitely not true. Participants for whom these beliefs were more important were more likely to prefer cardiopulmonary resuscitation (estimated probability for definitely true: 69.8% [95% CI, 66.5%-73.2%]; tends to be true: 60.8% [95% CI, 53.4%-68.3%]; tends not to be true: 61.6% [95% CI, 53.6%-69.6%]; and definitely not true: 60.6% [95% CI, 52.5%-68.6%]; *P* for trend = .003) and mechanical ventilation (estimated probability for definitely true: 42.6% [95% CI, 38.1%-47.0%]; tends to be true: 33.5% [95% CI, 25.9%-41.2%]; tends not to be true: 35.1% [95% CI, 27.2%-42.9%]; and definitely not true: 27.9% [95% CI, 19.6%-36.1%]; *P* for trend = .002) and to prefer a shared role in decision-making (estimated probability for definitely true: 41.6% [95% CI, 37.7%-45.5%]; tends to be true: 35.4% [95% CI, 29.0%-41.8%]; tends not to be true: 36.0% [95% CI, 26.7%-45.2%]; and definitely not true: 23.8% [95% CI, 17.3%-30.3%]; *P* for trend = .001) and were less likely to have thought or spoken about stopping dialysis. These participants were no less likely to have engaged in advance care planning, to value relief of pain and discomfort, to prefer to die at home, to have ever thought or spoken about hospice, and to have unmet palliative care needs and had similar prognostic expectations.

**Conclusions and Relevance:**

The finding that religious or spiritual beliefs were important to most study participants suggests the value of an integrative approach that addresses these beliefs in caring for people who receive dialysis.

## Key Points

**Question:**

Is there an association between the self-reported importance of religious or spiritual beliefs and serious illness preferences among people who receive dialysis?

**Findings:**

In this cross-sectional survey study of 937 patients receiving dialysis, most participants indicated that their religious or spiritual beliefs were behind their whole approach to life. Those for whom these beliefs were more important were more likely to favor resuscitation and a shared (vs patient-centered) decision-making role and less likely to have ever thought or spoken about stopping dialysis.

**Meaning:**

These findings highlight the importance of religious or spiritual beliefs and the potential value of an integrative approach that includes spiritual care for people who receive dialysis.

## Teaser

This survey study explores the advance care planning choices among adults receiving maintenance dialysis who hold religious or spiritual beliefs.

## Introduction

Spirituality, defined as “the way individuals seek and express meaning and purpose and the way they experience their connectedness to the moment, to self, to others, to nature, and to the significant or sacred,”^1^^(p887)^ is an important dimension of overall wellness. Among those with underlying health conditions, spirituality has been associated with existential wellness and with the psychological and material experiences of illness.^2^ Spirituality can help patients cope with serious illness and has been associated with better quality of life, whereas spiritual distress can contribute to feelings of hopelessness and depression.^3,4,5,6^ An understanding of patients’ religious and spiritual beliefs can be especially helpful in planning for serious illness, dealing with health challenges, and negotiating difficult treatment decisions at the end of life.^7,8^ Spiritual assessment is a core domain of palliative care, a medical specialty that supports people with serious illness.^9^

Although people receiving maintenance dialysis have lower life expectancy and a higher burden of comorbidity compared with those with normal kidney function, relatively few studies have examined spirituality and religious beliefs among members of this population. Previous studies on this topic have suggested that patients with advanced kidney disease have a substantial number of unmet spiritual needs^10,11,12^ and that higher levels of spirituality are associated with better quality of life.^13^ However, these previous studies offer few insights into the role of religious or spiritual beliefs in shaping patients’ approach to serious illness. Among patients with cancer or chronic illness, spirituality or reliance on religious coping has been associated with a greater desire for life-extending interventions (particularly among racial/ethnic minority groups),^14,15,16^ a lower likelihood of depression,^17^ a lower overall symptom burden,^18^ and less engagement in advance care planning.^19^

In this study, we aimed to examine whether there is an association between the importance of religious or spiritual beliefs and care preferences and palliative care needs in people who receive dialysis. We hypothesized that most patients receiving maintenance dialysis would view religious or spiritual beliefs as important and that those for whom these beliefs are more important would be more likely to value life extension over relief of pain and discomfort, to favor aggressive life-prolonging treatments, and to have more optimistic prognostic expectations and would be less likely to engage in advance care planning and to have unmet palliative care needs.

## Methods

### Design, Setting, and Participants

We conducted a cross-sectional survey study as part of the United States Renal Data System Study of Treatment Preferences (USTATE).^20,21,22^ The study was approved by the institutional review board at the University of Washington in Seattle, Washington. All participants provided written informed consent. We followed the Strengthening the Reporting of Observational Studies in Epidemiology (STROBE) reporting guideline.^23^

The USTATE survey was administered to patients who were receiving maintenance dialysis at 31 nonprofit dialysis facilities in Seattle, Washington, and Nashville, Tennessee, from April 22, 2015, to October 2, 2018. The survey included a series of questions assessing patients’ knowledge, preferences, values, and expectations related to end-of-life care. To be eligible to participate in the survey, patients had to be at least 21 years of age, sufficiently fluent in English to complete the survey, and cognitively able to provide written informed consent. Study staff consulted with dialysis facility charge nurses to identify patients who met the eligibility criteria, and then approached eligible patients during their dialysis sessions to invite them to participate in the study. This process yielded a pragmatic consecutive sample of eligible patients who were receiving maintenance dialysis at participating facilities at the time of survey administration. Most patients were receiving in-center hemodialysis, but a small convenience sample was receiving peritoneal dialysis. Participants could choose to complete the paper survey themselves or to have a study coordinator record their verbal responses.

After an initial pilot phase, 1431 eligible patients were invited to participate in the survey, of whom 997 (69.7%) provided written informed consent and completed the survey. We excluded those who were missing information on the importance of religious or spiritual beliefs or any of the self-reported baseline characteristics that were included in the multivariable analyses, which yielded an analytic cohort of 937 participants or 65.5% of those who were invited to participate.

### Exposure, Covariates, and Outcomes

The importance of religious or spiritual beliefs was ascertained using a single item from the Duke University Religion Index.^24^ Study participants were asked to respond to the following statement: “My religious or spiritual beliefs are what really lie behind my whole approach to life.” Possible responses included definitely true, tends to be true, tends not to be true, and definitely not true.^24^

Multivariate analyses included the following self-reported participant characteristics ascertained at the time of survey administration that we postulated might be associated with the importance of religious or spiritual beliefs: age (<60, 60-74, or ≥75 years), sex, self-reported race (White, Black, or other, which included Asian, American Indian or Alaskan Native, and Native Hawaiian or other Pacific Islander), Hispanic ethnicity, self-rated health status (excellent or very good, good, or fair or poor), highest educational level (<high school, graduated from high school or earned a GED [General Educational Development] certificate, attended some college or trade school, graduated from college or trade school, or received some postgraduate training), time since starting dialysis (<1, 1-5, or >5 years), and recruitment site (Nashville, Tennessee, or Seattle, Washington). For descriptive purposes, we included self-reported religious affiliation (Christian, other [Buddhist, Muslim, Jewish, or other], none, or missing information), but unlike other baseline characteristics, this variable was not included in the multivariable analyses.

Outcome measures included in the current analysis were based on responses to survey questions about (1) documentation of a surrogate decision-maker; (2) documentation of treatment preferences; (3) preference for cardiopulmonary resuscitation (CPR), with response options of definitely wanted to receive CPR, probably, probably not, or definitely not^20^; (4) preference for mechanical ventilation, with response options of definitely wanted to receive mechanical ventilation, probably, probably not, or definitely not^20^; (5) values regarding life prolongation, with response options of extending life, relieving pain and discomfort, or not sure; (6) preference for place of death, with response options of home or home of a relative or friend or other setting; (7) preference for decision-making role, with response options of patient-centered, shared, or physician-centered; (8) previous thoughts or discussion about stopping dialysis; (9) previous thoughts or discussion about hospice; and (10) prognostic expectations, with response options of less than 5, 5 to 10, or more than 10 years or not sure (Table 1). We also examined the association of self-reported importance of religious or spiritual beliefs with 18 different palliative care needs.^11^

**Table 1.** Characteristics of United States Renal Data System Study of Treatment Preferences (USTATE) Participants Included in the Analytic Sample

USTATE participant characteristic	No. (%)					*P* value
	All participants (n = 937)	Response to statement about importance of religious or spiritual beliefs				
		Definitely true (n = 435)	Tends to be true (n = 230)	Tends not to be true (n = 137)	Definitely not true (n = 135)	
Age group, y						.006
<60	372 (39.7)	168 (38.6)	81 (35.2)	52 (38.0)	71 (52.6)	
60-74	388 (41.4)	193 (44.4)	93 (40.4)	54 (39.4)	48 (35.6)	
≥75	177 (18.9)	74 (17.0)	56 (24.3)	31 (22.6)	16 (11.9)	
Sex						<.001
Female	413 (44.1)	225 (51.7)	101 (43.9)	49 (35.8)	38 (28.1)	
Male	524 (55.9)	210 (48.3)	129 (56.1)	88 (64.2)	97 (71.9)	
Race						<.001
White	558 (59.6)	215 (49.4)	143 (62.2)	103 (75.2)	97 (71.9)	
Black	254 (27.1)	160 (36.8)	56 (24.3)	14 (10.2)	24 (17.8)	
Other^a^	125 (13.3)	60 (13.8)	31 (13.5)	20 (14.6)	14 (10.4)	
Ethnicity						.50
Non-Hispanic	884 (94.3)	415 (95.4)	214 (93.0)	127 (92.7)	128 (94.8)	
Hispanic	53 (5.7)	20 (4.6)	16 (7.0)	10 (7.3)	7 (5.2)	
Self-rated health status						.07
Very good or excellent	182 (19.4)	87 (20.0)	55 (23.9)	27 (19.7)	13 (9.6)	
Good	358 (38.2)	168 (38.6)	84 (36.5)	49 (35.8)	57 (42.2)	
Fair or poor	397 (42.4)	180 (41.4)	91 (39.6)	61 (44.5)	65 (48.1)	
Highest educational level						.87
<High school	114 (12.2)	52 (12.0)	33 (14.3)	16 (11.7)	13 (9.6)	
Graduated from high school^b^	309 (33.0)	147 (33.8)	74 (32.2)	39 (28.5)	49 (36.3)	
Attended some college or trade school	164 (17.5)	78 (17.9)	36 (15.7)	26 (19.0)	24 (17.8)	
Graduated from college or trade school	296 (31.6)	134 (30.8)	76 (33.0)	48 (35.0)	38 (28.1)	
Received some postgraduate training	54 (5.8)	24 (5.5)	11 (4.8)	8 (5.8)	11 (8.1)	
Time since starting dialysis, y						<.001
<1	268 (28.6)	96 (22.1)	68 (29.6)	55 (40.1)	49 (36.3)	
1-5	449 (47.9)	219 (50.3)	103 (44.8)	62 (45.3)	65 (48.1)	
>5	220 (23.5)	120 (27.6)	59 (25.7)	20 (14.6)	21 (15.6)	
Recruitment site						<.001
Nashville, Tennessee	229 (24.4)	143 (32.9)	59 (25.7)	13 (9.5)	14 (10.4)	
Seattle, Washington	708 (75.6)	292 (67.1)	171 (74.3)	124 (90.5)	121 (89.6)	
Religion						<.001
Christian	704 (75.1)	379 (87.1)	188 (81.7)	92 (67.2)	45 (33.3)	
Other^c^	79 (8.4)	39 (9.0)	21 (9.1)	14 (10.2)	5 (3.7)	
None	128 (13.7)	7 (1.6)	10 (4.3)	29 (21.2)	82 (60.7)	
Missing information	32 (3.4)	12 (2.8)	14 (6.1)	2 (1.5)	4 (3.0)	

^a^ Other race included Asian, American Indian or Alaskan Native, and Native Hawaiian or other Pacific Islander.

^b^ Or earned a General Educational Development (GED) certificate.

^c^ Other religion included Buddhist, Muslim, Jewish, or other. Some participants selected more than 1 religious affiliation.

### Statistical Analyses

We used a χ^2^ test to describe the characteristics of participants with differing responses to the statement about religious or spiritual beliefs. We used logistic and multinomial regression as appropriate to estimate odds ratios for the association of the exposure variable with each outcome after adjustment for the aforementioned covariates and clustered by dialysis facility. The results of adjusted analyses are presented herein as estimated probabilities with 95% CIs, which were based on fixing the value of the adjustment variables at the mean value for the analytic cohort. The statistical significance of the adjusted associations with each outcome was assessed by testing for linear trends across the 4 categories of the exposure variable. Analyses for individual outcomes were restricted to participants with complete information on the relevant outcome, which ranged from 915 to 937 participants across outcome measures (Table 2 and Table 3).

**Table 2.** Adjusted Association Between Importance of Religious or Spiritual Beliefs and Domains of End-of-Life Care^a^

End-of-life care domain	No. of responses/total No. of responses (unadjusted %)	Response to statement about importance of religious or spiritual beliefs, estimated probability (95% CI)				*P* value for trend
		Definitely true (n = 435)	Tends to be true (n = 230)	Tends not to be true (n = 137)	Definitely not true (n = 135)	
Documented surrogate decision-maker	454/927 (49.0)	48.7 (44.1-53.2)	47.2 (40.1-54.4)	52.0 (42.8-61.2)	49.9 (41.6-58.2)	.64
Documented treatment preferences	351/934 (37.6)	38.8 (33.2-44.3)	34.1 (27.5-40.6)	38.7 (31.8-45.6)	38.7 (31.2-46.3)	.95
Preference for CPR	609/937 (65.0)	69.8 (66.5-73.2)	60.8 (53.4-68.3)	61.6 (53.6-69.6)	60.6 (52.5-68.6)	.003
Preference for mechanical ventilation	344/926 (37.1)	42.6 (38.1-47.0)	33.5 (25.9-41.2)	35.1 (27.2-42.9)	27.9 (19.6-36.1)	.002
Values						
Life prolongation	182/932 (19.5)	21.5 (17.7-25.2)	16.8 (12.4-21.1)	17.9 (11.0-24.9)	19.1 (13.8-24.4)	.40
Relief of pain and discomfort	450/932 (48.3)	47.7 (42.4-52.9)	51.7 (47.0-56.3)	43.5 (35.4-51.7)	49.9 (39.7-60.1)	.96
Not sure	300/932 (32.2)	30.9 (26.9-34.9)	31.5 (27.1-36.0)	38.5 (31.8-45.3)	31.0 (23.3-38.7)	.50
Preference for place of death: at home or in the home of a relative or friend	545/918 (59.4)	58.8 (54.6-63.0)	61.7 (55.7-67.7)	56.8 (47.2-66.4)	59.7 (51.4-68.1)	.96
Preference for decision-making						
Patient-centered	472/915 (51.6)	48.1 (42.4-53.7)	50.9 (44.9-56.9)	52.0 (44.3-59.8)	63.5 (57.3-69.6)	.006
Shared	336/915 (36.7)	41.6 (37.7-45.5)	35.4 (29.0-41.8)	36.0 (26.7-45.2)	23.8 (17.3-30.3)	.001
Physician-centered	107/915 (11.7)	10.3 (6.5-14.1)	13.7 (9.0-18.4)	12.0 (6.4-17.6)	12.7 (6.1-19.3)	.41
Ever thought about stopping dialysis	289/936 (30.9)	25.6 (21.5-29.7)	34.0 (28.7-39.2)	34.9 (25.8-44.1)	38.1 (29.8-46.4)	.004
Ever spoken with someone about stopping dialysis	258/937 (27.5)	23.7 (20.3-27.0)	29.4 (22.8-36.0)	30.7 (23.8-37.6)	33.6 (27.0-40.2)	.005
Ever thought about whether you would want hospice	500/933 (53.6)	54.6 (50.1-59.1)	56.4 (48.1-64.7)	51.2 (42.6-59.8)	47.9 (39.1-56.7)	.17
Ever spoken with someone about hospice	216/933 (23.2)	25.2 (21.2-29.2)	23.4 (16.2-30.7)	18.4 (13.7-23.1)	20.4 (15.0-25.9)	.10
Prognostic expectations, y						
<5	108/934 (11.6)	8.7 (6.5-10.8)	13.7 (9.1-18.4)	14.1 (7.7-20.5)	13.6 (8.2-19.1)	.051
5-10	142/934 (15.2)	17.8 (14.2-21.5)	12.2 (8.5-15.9)	16.7 (9.8-23.4)	10.8 (5.0-16.6)	.10
>10	310/934 (33.2)	34.9 (30.0-38.7)	34.0 (28.4-39.6)	29.2 (22.9-35.4)	30.8 (23.2-38.5)	.23
Not sure	374/934 (40.0)	38.6 (33.6-43.6)	40.1 (33.8-46.4)	40.1 (31.2-49.1)	44.8 (35.1-54.4)	.22

Abbreviation: CPR, cardiopulmonary resuscitation.

^a^ Adjusted for age, sex, race, ethnicity, self-rated health status, highest educational level, time since starting dialysis, and recruitment site.

**Table 3.** Adjusted Association Between Importance of Religious or Spiritual Beliefs and Palliative Care Needs^a^

Survey question	No. of responses/total No. of responses (unadjusted %)	Response to statement about importance of religious or spiritual beliefs, estimated probability (95% CI)				*P* value for trend
		Definitely true (n = 435)	Tends to be true (n = 230)	Tends not to be true (n = 137)	Definitely not true (n = 135)	
**I would like to learn more about**						
How to be in touch with other patients with kidney disease	270/933 (28.9)	32.9 (27.0-38.7)	27.3 (20.9-33.8)	23.9 (15.3-32.5)	23.5 (17.4-29.6)	.01
What I can do about pain	425/936 (45.4)	48.0 (44.4-51.6)	44.3 (36.1-52.5)	45.4 (35.5-55.2)	38.9 (33.6-44.2)	.01
Relaxation or stress management	370/934 (39.6)	42.4 (36.9-47.9)	38.3 (31.9-44.7)	37.2 (28.1-46.2)	35.3 (25.1-45.5)	.18
Treating the symptoms of kidney disease	548/935 (58.6)	58.8 (54.3-62.3)	56.1 (49.4-62.8)	62.1 (54.4-69.9)	58.7 (52.0-65.5)	.78
**I would like help with**						
Making plans in case I become very ill	407/933 (43.6)	47.0 (42.8-51.3)	40.9 (35.2-46.6)	40.7 (29.5-52.0)	40.1 (31.3-48.9)	.10
Learning to cope with feelings of sadness	260/933 (27.9)	29.4 (24.1-34.7)	25.4 (18.6-32.3)	29.8 (22.2-37.4)	25.0 (18.2-31.8)	.52
Sharing my thoughts and feelings with those close to me	260/932 (27.9)	30.0 (24.4-35.7)	26.1 (20.5-31.6)	24.1 (18.4-29.9)	27.4 (21.7-33.1)	.23
Finding spiritual resources	139/931 (14.9)	16.2 (12.3-20.2)	15.9 (12.5-19.3)	10.8 (5.4-16.3)	11.3 (6.4-16.3)	.09
**I would like to have help with**						
Worries I have about the effect of my illness on my family	279/931 (30.0)	31.1 (27.6-34.6)	30.1 (24.9-35.4)	29.2 (19.6-38.7)	26.9 (18.0-35.8)	.35
Finding meaning in my life now	163/931 (17.5)	17.3 (13.3-21.7)	19.3 (14.4-24.2)	16.6 (8.1-25.1)	16.2 (10.8-21.5)	.82
Finding hope	197/928 (21.2)	23.3 (19.0-27.5)	21.9 (16.0-27.9)	15.6 (8.7-22.5)	18.1 (11.7-24.5)	.045
Overcoming my fears	186/932 (20.0)	21.5 (16.0-27.0)	15.6 (10.8-20.4)	22.5 (13.2-31.8)	19.4 (14.0-24.8)	.65
Organizing my appointments and treatments	231/929 (24.9)	24.9 (21.0-28.8)	24.9 (19.7-30.2)	26.6 (17.3-35.9)	23.1 (17.5-28.7)	.82
**I would like to have someone to talk to about**						
My care plan and treatments	300/932 (32.2)	32.0 (27.0-37.0)	34.9 (29.5-40.3)	32.1 (21.8-42.4)	28.4 (21.9-34.8)	.57
Treatment options for the future	452/933 (48.4)	51.4 (46.6-56.1)	51.5 (44.9-58.0)	45.9 (34.4-57.3)	37.0 (30.0-44.0)	.01
The meaning of life	115/929 (12.4)	13.2 (10.4-16.0)	11.8 (6.9-16.8)	13.5 (7.0-20.1)	9.0 (4.1-13.9)	.34
Dying and death	120/931 (12.9)	13.4 (9.5-17.4)	12.2 (8.4-16.1)	11.9 (6.2-17.6)	13.0 (8.0-18.0)	.77
Finding peace of mind	194/932 (20.8)	21.0 (16.9-25.1)	23.7 (17.4-30.1)	21.0 (11.8-30.3)	15.0 (8.9-21.2)	.27

^a^ Adjusted for age, sex, race, ethnicity, self-rated health status, highest educational level, time since starting dialysis, and recruitment site.

A 2-sided *P* < .05 indicated statistical significance. All analyses were conducted with Stata, version 16 (StataCorp LLC). Data were analyzed from February 12, 2020, to April 21, 2021.

## Results

### Study Participants

Of the 937 USTATE participants included in the analytic sample, the mean (SD) age was 62.8 (13.8) years, 524 (55.9%) were men, 413 (44.1%) were women, 254 (27.1%) self-reported as Black individuals, and 53 (5.7%) self-reported as Hispanic individuals (Table 1). A total of 182 participants (19.4%) rated their own health as very good or excellent, 358 (38.2%) rated their health as good, and 397 (42.4%) rated their health as fair or poor. A total of 114 participants (12.2%) did not complete high school, 309 (33.0%) graduated from high school or obtained a GED equivalency certificate, 164 (17.5%) attended some college or trade school, 296 (31.6%) graduated from college or trade school, and 54 (5.8%) received some postgraduate education.

Overall, 268 participants (28.6%) had been receiving dialysis for less than a year, 449 (47.9%) had received it for between 1 and 5 years, and 220 (23.5%) had received it for more than 5 years. Most participants (708 [75.6%]) resided in Seattle, Washington, and the rest of the sample was located in Nashville, Tennessee (229 [24.4%]). Most participants (704 [75.1%]) described themselves as Christian, 79 (8.4%) listed other religious affiliations, 128 (13.7%) reported no religious affiliation, and 32 (3.40%) did not respond to the question on religious affiliation.

Among the 937 participants, 435 (46.4%) rated the statement “My religious or spiritual beliefs are what really lie behind my whole approach to life,” as definitely true, 230 (24.6%) rated it as tends to be true, 137 (14.6%) rated it as tends not to be true, and 135 (14.4%) rated it as definitely not true; the first 2 categories represented a total of 665 participants (70.1%). Older participants, women (vs men), those who self-identified as Black (vs White) individuals, those who had been receiving dialysis for a longer time (1-5 or >5 years), and those who were recruited from Nashville, Tennessee, were more likely to agree that their spiritual beliefs were important to them (Table 1). Participants who self-identified as Christian were more likely and those who reported no religious affiliation were less likely to agree that religious or spiritual beliefs were important to them.

### Association of the Importance of Religious or Spiritual Beliefs With Domains of End-of-Life Care

In analyses that were adjusted for age group, sex, race, ethnicity, self-rated health status, highest educational level, time since starting dialysis, and recruitment site, participants who reported that religious or spiritual beliefs were important were more likely to have a preference for CPR (estimated probability for definitely true: 69.8% [95% CI, 66.5%-73.2%]; tends to be true: 60.8% [95% CI, 53.4%-68.3%]; tends not to be true: 61.6% [95% CI, 53.6%-69.6%]; and definitely not true: 60.6% [95% CI, 52.5%-68.6%]; *P* for trend = .003) and mechanical ventilation (estimated probability for definitely true: 42.6% [95% CI, 38.1%-47.0%]; tends to be true: 33.5% [95% CI, 25.9%-41.2%]; tends not to be true: 35.1% [95% CI, 27.2%-42.9%]; and definitely not true: 27.9% [95% CI, 19.6%-36.1%]; *P* for trend = .002). In addition, this group was more likely to prefer shared decision-making (estimated probability for definitely true: 41.6% [95% CI, 37.7%-45.5%]; tends to be true: 35.4% [95% CI, 29.0%-41.8%]; tends not to be true: 36.0% [95% CI, 26.7%-45.2%]; and definitely not true: 23.8% [95% CI, 17.3%-30.3%]; *P* for trend = .001), was less likely to prefer patient-centered decision-making (estimated probability for definitely true: 48.1% [95% CI, 42.4%-53.7%]; tends to be true: 50.9% [95% CI, 44.9%-56.9%]; tends not to be true: 52.0% [95% CI, 44.3%-59.8%]; and definitely not true: 63.5% [95% CI, 57.3%-69.6%]; *P* for trend = .006), and was less likely to have thought about stopping dialysis (estimated probability for definitely true: 25.6% [95% CI, 21.5%-29.7%]; tends to be true: 34.0% [95% CI, 28.7%-39.2%]; tends not to be true: 34.9% [95% CI, 25.8%-44.1%]; and definitely not true: 38.1% [95% CI, 29.8%-46.4%]; *P* = .004) or spoken about stopping dialysis (estimated probability for definitely true: 23.7% [95% CI, 20.3%-27.0%]; tends to be true: 29.4% [95% CI, 22.8%-36.0%]; tends not to be true: 30.7% [95% CI, 23.8%-37.6%]; and definitely not true: 33.6% [95% CI, 27.0%-40.2%]; *P* for trend = .005) (Table 2).

No statistically significant trends were found across responses to the statement about the importance of religious or spiritual beliefs under the end-of-life care domains of documentation of a surrogate decision-maker or treatment preferences, preference for place of death, values regarding life prolongation vs relief of pain and discomfort, prognostic expectations, or previous thoughts or discussions about hospice.

### Association of Importance of Religious or Spiritual Beliefs With Palliative Care Needs

In adjusted analyses, participants for whom religious or spiritual beliefs were important were no less likely to have unmet palliative care needs. They were more likely to want to learn about how to be in touch with other patients with kidney disease (estimated probability for definitely true: 32.9% [95% CI, 27.0%-38.7%]; tends to be true: 27.3% [95% CI, 20.9%-33.8%]; tends not to be true: 23.9% [95% CI, 15.3%-32.5%]; and definitely not true: 23.5% [95% CI, 17.4%-29.6%]; *P* for trend = .01) and what they can do about pain (estimated probability for definitely true: 48.0% [95% CI, 44.4%-51.6%]; tends to be true: 44.3% [95% CI, 36.1%-52.5%]; tends not to be true: 45.4% [95% CI, 35.5%-55.2%]; and definitely not true: 38.9% [95% CI, 33.6%-44.2%]; *P* for trend = .01). In addition, these participants were more likely to want help with finding hope (estimated probability for definitely true: 23.3% [95% CI, 19.0%-27.5%]; tends to be true: 21.9% [95% CI, 16.0%-27.9%]; tends not to be true: 15.6% [95% CI, 8.7%-22.5%]; and definitely not true: 18.1% [95% CI, 11.7%-24.5%]; *P* for trend = .045) and to want someone to talk with about treatment options for the future (estimated probability for definitely true: 51.4% [95% CI, 46.6%-56.1%]; tends to be true: 51.5% [95% CI, 44.9%-58.0%]; tends not to be true: 45.9% [95% CI, 34.4%-57.3%]; and definitely not true: 37.0% [95% CI, 30.0%-44.0%]; *P* for trend = .01) (Table 3).

## Discussion

To our knowledge, this study is the first to describe the association between the self-reported importance of religious or spiritual beliefs and knowledge, expectations, values, and preferences related to serious illness and palliative care needs of a cohort of patients who were receiving maintenance dialysis. Religious or spiritual beliefs were at least of some importance to most participants (70.1%), a finding that is consistent with results from previous work in various other populations with chronic illness, among whom the proportion who identified as religious or spiritual has ranged from 19% to 84%.^4,12,14,25,26^

As we hypothesized and as consistent with the results of studies conducted in other populations,^14,19^ the USTATE participants for whom religious or spiritual beliefs were more important were more likely to favor the use of life-extending interventions, such as CPR and mechanical ventilation. These respondents were also less likely to have ever thought or spoken about stopping dialysis and more likely to favor a shared decision-making role over a patient-centered decision-making role. Despite a preference for life-prolonging treatments, these participants were no less likely to have engaged in advance care planning or to value relief of pain and discomfort. These findings suggest that understanding the religious or spiritual beliefs of people who receive dialysis could be helpful in framing the discussions about their wishes for future care and understanding treatment preferences.

Studies in populations who are not receiving dialysis have suggested that religious or spiritual beliefs (as well as related concepts, such as finding hope, meaning, and a sense of peace) can help patients cope with advanced illness.^17,27,28^ However, in this study, we found that participants for whom religious or spiritual beliefs were more important were no less likely to have unmet palliative care needs and were more likely to report needs related to peer support, pain management, finding hope, and learning about treatment options for the future. Although some evidence suggests that incorporating spiritual care into other aspects of medical care can help support and shape patients’ treatment decisions,^14,29^ spirituality is rarely addressed in real-world clinical settings, even among seriously ill patients. One study found that fewer than 20% of goals-of-care discussions with surrogate decision-makers of patients in the intensive care unit addressed spiritual or religious beliefs.^30^ Furthermore, when spiritual concerns were raised by surrogate decision-makers, clinicians tended to respond by redirecting the conversation toward medical matters.^30^ Substantial differences in treatment preferences among participants with differing responses to the statement about the importance of spiritual or religious beliefs, despite having similar exposure to advance care planning and similar life-prolongation values, highlight the potential advantage of integrating spiritual care into advance care planning and other aspects of care for people who receive dialysis.

The finding that religious or spiritual beliefs were more important to female (vs male) and to Black (vs White) participants is consistent with the results of previous studies both among people who receive dialysis and in more broadly defined populations.^12,13,16,26,31,32,33,34,35,36,37^ For example, in a study of 165 people on hemodialysis, Kimmel et al^13^ found that spiritual belief scores were substantially higher for women than for men. Among 51 Black patients on hemodialysis, Song and Hanson^12^ found that more than half (61%) reported that spirituality was important to them; among 166 predominantly Black patients who were undergoing hemodialysis, Spinale et al^34^ found that scores of spirituality, defined as a perception of the importance of faith and the role it plays in coping with kidney disease, were high. Along with results from these earlier studies, the findings in this study suggest that an integrative approach to care that addresses the religious and/or spiritual beliefs of people who receive dialysis may be particularly beneficial for women and Black individuals. In addition, these findings suggest that the value of integrating spiritual beliefs into the care of people who receive dialysis may be greater for older individuals and those who have been receiving dialysis for a longer time and may vary geographically.

### Limitations

This study has several limitations. First, the study question examined only the importance of religious or spiritual beliefs and did not explore the more specific concepts of spiritual well-being and distress, nor did the question distinguish between spirituality and religious beliefs. Although often conflated with spirituality, religion is a distinct concept, defined as an “organized system of beliefs, practices, and symbols designed to facilitate closeness to the transcendent or the Divine and foster an understanding of one’s relationship and responsibilities with others living in a community.”^8^^(p429)^ The results of this study do not account for the possibility that these separate constructs may have distinct associations with the outcome measures we evaluated. Second, the exclusion criteria and the composition of the cohort (predominantly English-speaking individuals who were receiving in-center hemodialysis at nonprofit facilities in Seattle, Washington, or Nashville, Tennessee) may limit the generalizability of the findings to other segments of the dialysis population. Sizeable differences in the importance of religious or spiritual beliefs between USTATE participants who were recruited from these 2 metropolitan areas suggest the potentially limited generalizability of the findings to other parts of the United States. Some of the outcome measures we examined may also be sensitive to regional differences in practice (eg, engagement in advance care planning), further limiting the generalizability of the results. Third, because of the cross-sectional observational design of the study, the associations we described herein cannot be interpreted as causal.

## Conclusions

We found that religious or spiritual beliefs were at least of some importance to most USTATE participants who were receiving maintenance dialysis. The importance of these beliefs was associated with several domains of end-of-life care planning, including resuscitation preferences, thoughts and discussions about stopping dialysis, and decision-making preferences. These findings suggest the potential value of an integrative approach (that addresses religious and/or spiritual beliefs) to caring for members of this population.
